# Revisiting risk-profiling in pediatric cardiac anesthesia: a commentary on troponin-based outcome measures*

**DOI:** 10.1016/j.bjane.2025.844634

**Published:** 2025-05-02

**Authors:** Rohan Magoon

**Affiliations:** Atal Bihari Vajpayee Institute of Medical Sciences (ABVIMS) and Dr. Ram Manohar Lohia Hospital, Department of Cardiac Anaesthesia, Baba Kharak Singh Marg, India

Dear Editor,

The recently published Randomized Clinical Trial (RCT) by Barelli et al., has been read with great interest.^1^ The authors evaluate the impact of anesthetic technique on the troponin I levels, following pediatric cardiac surgery.^1^ Meanwhile the authors did not discover significant differences between the patients randomized to receive sevoflurane (n = 33) or total intravenous anesthesia (n = 33), the research subject, nonetheless, merits further discussion.^1^, ^2^, ^3^ First, the RCT would have been served well with the inclusion of details on the performance of Modified Ultrafiltration (MUF) in the study participants.^1^^,^^2^ The former becomes important considering independent researchers like Talwar et al. outline lower postoperative troponin T-levels following intracardiac repair, in backdrop of MUF being combined with conventional ultrafiltration in their patients with tetralogy of Fallot.^2^ Second, ahead of arrythmias in general, outcomes like junctional ectopic tachycardia or JET, are specifically related to congenital cardiac surgery with an incidence of 5%‒11%.^3^^,^^4^ Of note, JET has also been linked to postoperative troponin elevation, in a clinical review by Alasti et al., especially relevant with the investigation frame extending into the postoperative phase for 48 h, as was the case in the Barelli et al. RCT.^1^^,^^3^ Finally, the authors do apprise the readership on the role of Low Cardiac Output Syndrome (LCOS) in the matter. That said, with a total of 14/66 i.e., 21.21% of the RCT participants landing into LCOS (05 and 09 in the sevoflurane group and the TIVA group, respectively), Barelli et al. should have outlined the corresponding definition employed to label LCOS in their patients.^1^ Herein, although the authors qualitatively report the vasopressor-inotropic use, a simultaneous account of the doses employed would have enhanced the clarity and clinical relevance of their findings. In this context, the absence of Vasoactive-Inotropic Score (VIS) deserves attention.^1^ VIS is being increasingly recognized as a marker of postoperative cardiovascular support, as highlighted by the Pediatric Cardiac Critical Care Consortium.^5^

I trust the authors and readers will find these comments a constructive addition to the subject of risk-profiling in pediatric cardiac anesthesia, better envisaged as a perioperative continuum, right from the preoperative stratification to the operative and the anesthetic conduct, eventually leading on to the postoperative outcomes.

## Authors' contributions

Rohan Magoon: Literature search and manuscript preparation.

## Financial support

None.

## Declaration of competing interest

The authors declare no have conflicts of interest.
